# The functions and roles of C2H2 zinc finger proteins in hepatocellular carcinoma

**DOI:** 10.3389/fphys.2023.1129889

**Published:** 2023-06-29

**Authors:** Qinguo Li, Guoqian Tan, Fan Wu

**Affiliations:** Department of General Surgery, Guangzhou Red Cross Hospital Affiliated to Jinan University, Guangzhou, China

**Keywords:** C2H2 zinc finger proteins, hepatocellular carcinoma, EMT, stemness maintenance, metabolic reprogramming, anti-tumor drug resistance

## Abstract

C2H2 zinc finger (C2H2-ZF) proteins are the majority group of human transcription factors and they have many different molecular functions through different combinations of zinc finger domains. Hepatocellular carcinoma (HCC) is one of the most prevalent malignant tumors and the main reason for cancer-related deaths worldwide. More and more findings support the abnormal expression of C2H2-ZF protein in the onset and progression of HCC. The C2H2-ZF proteins are involved in various biological functions in HCC, such as EMT, stemness maintenance, metabolic reprogramming, cell proliferation and growth, apoptosis, and genomic integrity. The study of anti-tumor drug resistance also highlights the pivotal roles of C2H2-ZF proteins at the intersection of biological functions (EMT, stemness maintenance, autophagy)and chemoresistance in HCC. The involvement of C2H2-ZF protein found recently in regulating different molecules, signal pathways and pathophysiological activities indicate these proteins as the possible therapeutic targets, and diagnostic or prognostic biomarkers for HCC.

## 1 Introduction

Hepatocellular carcinoma (HCC) is a frequent malignant tumor with high morbidity and mortality and has been the third leading cause of cancer-related death worldwide (Sung et al., 2021). Treatment strategies for HCC require interdisciplinary and multidisciplinary approaches, including surgery, liver transplantation, tumor ablation, transcatheter arterial chemical embolization (TACE), interventional radiology, chemotherapy, tumor-targeted therapy, and immunotherapy. The clinical presentation of HCC has improved over the past 10 years with the emergence of adjuvant medications following surgical resection, ablation or chemoprevention (Villanueva, 2019). Although interventional therapy can dramatically improve patient prognosis when HCC is detected early, early detection of HCC is crucial for patients and highly related to patient prognosis, most patients with HCC are unfortunately diagnosed in the advanced phase, which reduces the likelihood of curable resection or liver transplantation. Systemic treatment is the most often used option for individuals with unresectable advanced HCC. Sorafenib, lenvatinib, regorafenib, cabozantinib, nivolumab, and ramucirumab are different drugs of targeted therapies that target cancer-promoting mechanisms at the level of gene expression (Llovet et al., 2018; Raoul et al., 2018). At present, there are no biomarkers that can satisfactorily predict the track of HCC, and no drugs can effectively target chemotherapy resistance or metastatic cells (Huang et al., 2020). Thus, understanding the potential progress of onset and development of HCC is very important in order to find more biomarkers and develop individualized treatment, which is beneficial to improve the prognosis of patients with HCC.

**FIGURE 1:**
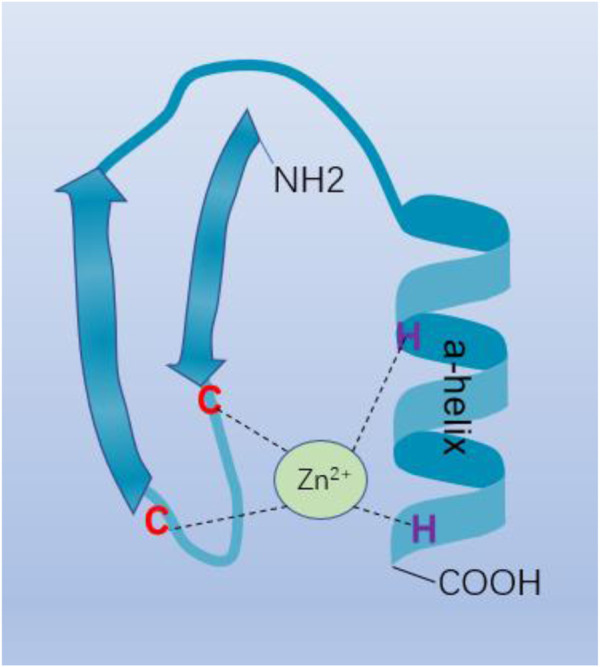
C2H2-type zinc finger motif. C2H2-ZF protein plays an important role in the binding of DNA sequences and conservative connectors between fingers through amino acid on the finger a-helix.

Transcription factors (TFs) play an important role in gene expression control by attaching to target DNA in a sequence-specific way. The Cys2-His2 (C2H2) zinc finger proteins represent the majority group of human TFs (Lander et al., 2001; Urrutia, 2003), almost half of which mainly bind to endogenous retroelements (EREs) (Najafabadi et al., 2015). C2H2 zinc finger (C2H2-ZF) protein is a transcription factor protein through a variety of structural shapes and motifs, with a variety of different molecular functions, including protein-protein or protein-RNA interaction, RNA binding and sequence-specific binding to DNA involved in transcriptional regulation (Stubbs et al., 2011; Weirauch and Hughes, 2011). The structure of the C2H2-ZF protein consists of a C-terminal DNA-binding C2H2-ZF domain and an N-terminal effect domain. These effect domains include POZ domain, also known as BTB domain, KRAB (Krüppel-Associated Box) domain, SCAN (SRE-ZBP, CTfin51, AW-1, and Number 18 cDNA) domain and SET {suppressor of variegation 3 to 9 [Su(var)3–9], enhancer of zeste [E(z)], and trithorax (Tx)} (Laity et al., 2001) domain. C2H2-ZF protein plays a key role in the binding of DNA sequences and conservative connectors between fingers through amino acid on the finger a-helix (Figure 1) (Lee et al., 1989). The BTB/POZ domain is a protein-protein interaction motif that has been conserved throughout evolution. The BTB (BroadComplex, Tamarack, and Bric-a-brac) domain improves the particularity and affinity of DNA binding by interacting with dimerization or its functional contact with genetic code areas. It also inhibits transcription by interacting with other families of proteins (BCL-6/PLZF) (Melnick et al., 2002). The KRAB domain, as well as BTB/POZ domain, plays a role in the transcriptional inhibition module, which recruits its cofactor TRIM28 to participate in silencing foreign retroviruses and ERE (Rowe and Trono, 2011). SCAN domain is a leucine-rich domain. Like the BTB/POZ domain, the SCAN box can mediate homologous and heterologous dimerization between certain SCAN domain family members (Collins et al., 2001). Some of these domains are related to the transcriptional control of genes involved in lipid metabolism, growth factors, and other genes essential for cell maintenance and development (Sander et al., 2003). The proteins containing the SET domain are part of a class of enzymes with the same common domain that methylate their substrate by using the cofactor S-adenosine-L-methionine (SAM). Regulation of signal pathways, transcription factors, and tumor suppressors are formed by the methylation of histones or the targeting of non-histone substrates, and tissue homeostasis is maintained (Herz et al., 2013). Therefore, through different combinations of zinc finger domains, C2H2-ZF proteins could enhance different roles in regulation through diverse cellular environments. In this review, we have briefly introduced the network regulation and anti-tumor drug resistance mechanisms of C2H2-ZF proteins in HCC (Tables 1, 2), which not only shows that C2H2-ZF proteins play significant roles in the development of HCC, but also suggests them as potential therapeutic targets and HCC biomarkers with diagnostic or prognostic value.

**TABLE 1:** C2H2 zinc finger proteins upregulate in HCC.

Function	Name of C2H2-ZF protein	Expression	Mechanism/pathway	Refs
EMT	ZEB1	Upregulation	IGFBP2/p65/ZEB1/NF-B	Guo et al. (2020)
			TXNDC12/Wnt/β-Catenin/ZEB1	Yuan et al. (2020)
			YB1 and microprocessors/miR-205,200b/ZEB1	Liu et al. (2021)
			miR-369/ZEB1	Dong et al. (2021a)
			miR-708/ZEB1	Li et al. (2021a)
			DCAF15/ZEB1	Dong et al. (2021b)
			USP39 or TRIM26/ZEB1	Li et al. (2021b)
EMT and anti-tumor drug resistance	ZEB1	Upregulation	ZEB1/PKC	Sreekumar et al. (2019)
			SIAH1 and miR-3129–5p/ZEB1	Long et al. (2019)
	ZEB2	Upregulation	linac-ROR and miR-145/ZEB2	Li et al. (2017b)
			miR-212–3p/ZEB2	Yang et al. (2020)
	Slug	Upregulation	Slug/ABCB1,ABCG2	Hoshida et al. (2016), Karaosmanoglu et al. (2018)
	GLI1	Upregulation	GLI1, TAP1/HH signal	Zhou et al. (2020)
Stemness maintenance	ZNF687	Upregulation	ZNF687/MBD3/NuRD/BMI1,Oct4,Nanog	Zhang et al. (2017)
			ZNF687/Wnt/β-catenin	Zhang et al. (2017)
	ZEB1	Upregulation	miR-200b/BMI1 and ZEB1/CD13,CD24,EpCAM	Tsai et al. (2017)
	ZFX	Upregulation	ZFX/Nanog and SOX2	Lai et al. (2014)
			ZFX/Wnt/β-catenin/c-Myc and CylinD1	Wang et al. (2017)
Stemness maintenance and anti-tumor drug resistance	ZNF687	Upregulation	ZNF687/BMI1,Oct4,Nanog	Zhang et al. (2017)
	ZFX	Upregulation	ZFX/Nanog and SOX2	Lai et al. (2014)
Aerobic glycolysis	ZEB1	Upregulation	ZEB1/PFKM	Zhou et al. (2021)
proliferation and growth	ZNF384	Upregulation	ZNF384/CyclinD1/CDK4 or CDK6	He et al. (2019)
	ZNF143	Upregulation	ZNF143/MDIG/CDC6	Zhang et al. (2020)
	ZNF233	Upregulation	ZNF233/G1/S	Xie et al. (2018)
Anoikis	ZNF32	Upregulation	ZNF32/ROS,Src/FAK	Li et al. (2019)
Autophagy and anti-tumor drug resistance	ZNF263	Upregulation	ERS/ZNF263	Cui et al. (2020)

**TABLE 2:** C2H2 zinc finger proteins downregulate in HCC.

Function	Name of C2H2-ZF protein	Expression	Mechanism/pathway	Refs
EMT	KLF17	Downregulation	TGF-β/Smad/KLF17	Ali et al. (2015)
			miR-9/KLF17	Sun et al. (2013)
Stemness maintenance	ZBP-89	Downregulation	ZBP-89/Notch1/EpCAM and CD44	Wang et al. (2020)
	ZHX2	Downregulation	ZHX2/SOX2, Nanog and Oct4	Lin et al. (2020)
Stemness maintenance and anti-tumor drug resistance	ZHX2	Downregulation	ZHX2/SOX2, Nanog and Oct4	Lin et al. (2020)
Aerobic glycolysis	ZFP91(E3 ligase)	Downregulation	ZFP91/hnRNP A1/PKM1	Chen et al. (2020)
NAFLD-HCC	ZHX2	Downregulation	ZHX2/LPL	Wu et al. (2020)
			ZHX2/miR-24–3p/SREBP1c/DNL	Yu et al. (2020)
Apoptosis	ZNF307	Downregulation	ZNF307/caspase-3 and Bax,Bcl-2	Liang et al. (2017)
	KLF6(SV2)	Downregulation	KLF6(SV2)/P53/P21 and Bax	Hanoun et al. (2010)
	KLF9	Downregulation	KLF9/P53 (GC box)	Sun et al. (2014)

## 2 EMT in HCC

A change from the epithelial to the mesenchymal state known as the epithelial-mesenchymal transition (EMT) changes the adhesion molecules that the cell expresses, enabling it to take on a migratory and invasive activity (Nieto et al., 2016). EMT endows cells with the characteristics to migrate and invade, induces stem cells, inhibits cell death and senescence, and relates to autoimmunity. EMT occurs as a result of multiple signaling pathways that trigger the production of particular transcription factors known as EMT-TF (such as Snail, ZEB1, ZEB2, Slug, etc.).

### 2.1 ZEB1

**FIGURE 2:**
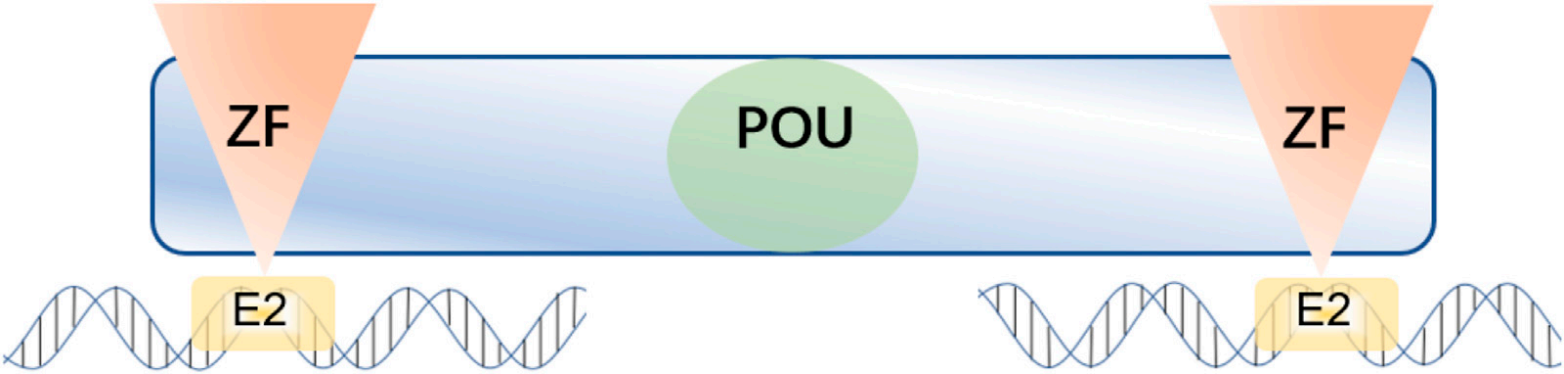
ZEB1 contains two C2H2-ZF domains at the C-terminal and N-terminal regions that bind to E2-box-like CACCT(G) sequences in the target gene promoter region, and a central POU-like homologous domain.

**FIGURE 3:**
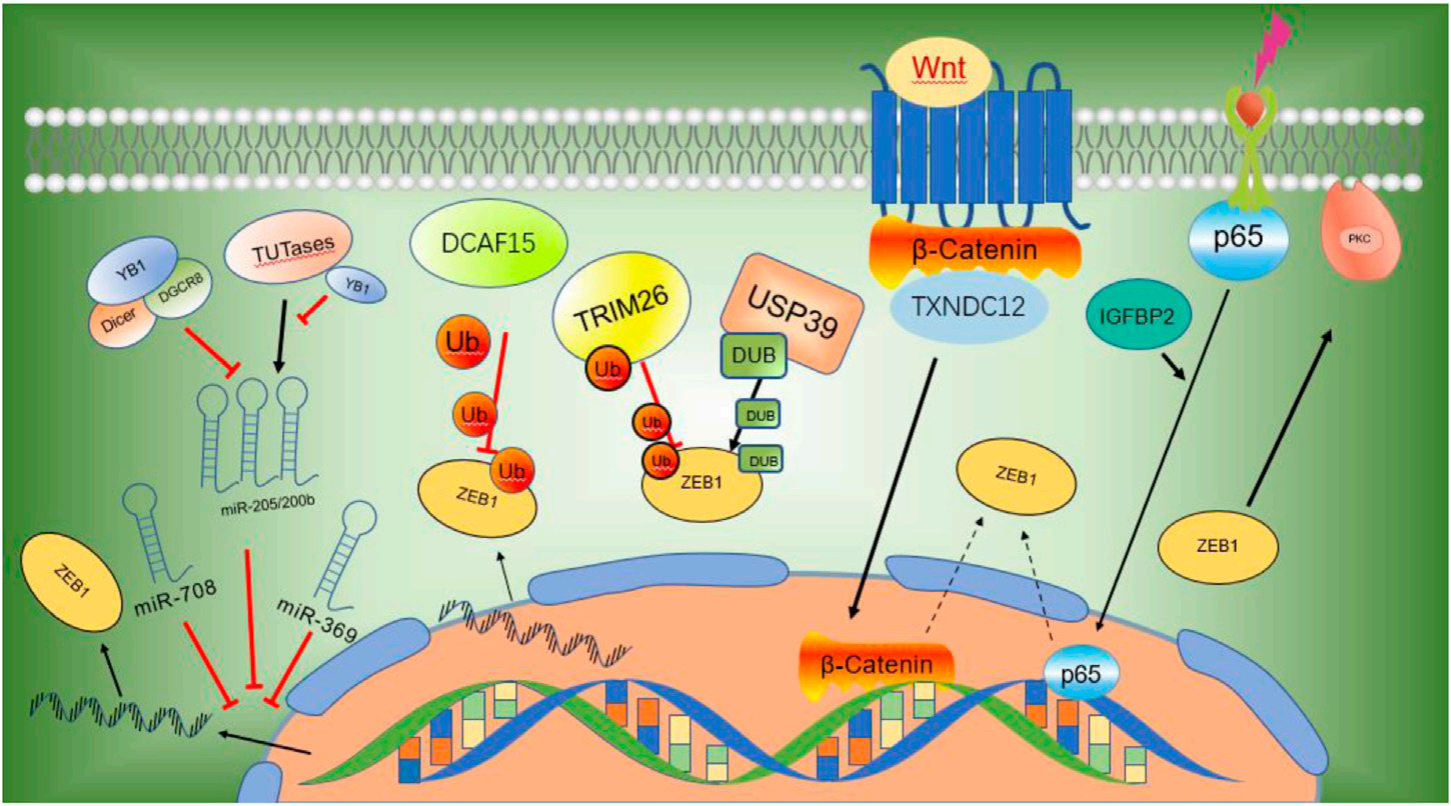
ZEB1 participates in different axis reactions through interacting with different molecules.

Zinc finger E-box-binding homeobox 1 (ZEB1) is an EMT-TF of the ZFH (zinc-finger-homeodomain) family. It contains two C2H2-ZF domains that interact with E2-box-like CACCT(G) motifs in the target gene regulatory regions and a central POU-like homologous domain that does not interact with DNA (Figure 2), which could mainly participate in protein-protein interactivity (Vandewalle et al., 2008). ZEB1 promotes EMT by inhibiting the expression of E-cadherin, which is a kind of cell adhesion molecule and inhibits tumorigenesis and invasion through the process of malignant transformation in HCC (Vandewalle et al., 2008). The downregulation of E-cadherin eventually leads to EMT, which is a key biological event in tumor cell metastasis. ZEB1 is highly expressed in HCC and its high expression is associated with clinical stage, morphological and pathological features of the tumor (including tumor size, intrahepatic metastasis, and vascular invasion) (Zhou et al., 2012). Insulin-like growth factor binding protein 2 (IGFBP2) promotes p65 nuclear translocation, which interacts with the promoter of ZEB1 and stimulates nuclear factor kappa B (NF-B) and ZEB1 expression in HCC (Guo et al., 2020). Moreover, thioredoxin domain-containing protein 12 (TXNDC12) increases the downstream ZEB1-mediated EMT process via activating Wnt/β-Catenin signal pathway to promote intimal metastasis and HCC metastasis (Yuan et al., 2020). ZEB1 participates in different axis reactions by interacting with different molecules (Figure 3).

#### 2.1.1 ZEB1 and miRNAs

MicroRNAs (miRNAs) have been demonstrated to suppress the expression of critical cancer-related genes and are implicated in the development and progression of human cancer (Calin and Croce, 2006). MicroRNAs exert inhibitory activity by interacting with ZEB1 mRNA on its 3′untranslated regions (UTRs), repressing ZEB1 expression post-transcriptionally or inducing mRNA degradation (Esquela-Kerscher and Slack, 2006). Y-box binding protein 1 (YB1) inhibits the maturation of miR-205 and miR-200b through binding to microprocessors or uridylyl transferases. The downregulation of miR-205 and miR-200b enhances ZEB1 transcription, resulting in enhanced cell movement and malignant transformation (Liu et al., 2021). Other studies have shown that miR-369 inhibits the expression of ZEB1 mRNA and protein by interacting with its 3′-UTR in HCC cells. MiR-369 highly expresses in HCC and inhibits EMT by promoting the expression of E-cadherin while decreasing the expression of the mesenchymal biomarkers vimentin and N-cadherin (Dong Y. et al., 2021). Li and colleagues reported that miR-708 could decrease the expression of ZEB1 by specifically interacting with ZEB1 mRNA on its 3′-UTR, and therefore inhibit the growth, proliferation, and metastasis of HCC through mediating Wnt/β-Catenin signal (Li LY. et al., 2021).

#### 2.1.2 Ubiquitination and deubiquitination of ZEB1

ZEB1 is used as the substrate of E3 ubiquitin ligase. Tumor suppressor genes DDB1 and CUL4 are the core components of CRL4-DCAF15 (DDB1 and CUL4 associated factor 15), E3 ubiquitin ligase, which specifically recognizes zinc finger domain on the N-terminal of ZEB1 and promotes ZEB1 ubiquitination and degradation, thereby suppressing cell growth, proliferation, and metastasis as well as EMT in HCC cells (Dong X. et al., 2021). Other studies have shown that Ubiquitin-specific peptidase 39 (USP39) deubiquitinates and suppresses ZEB1 degradation, thus promoting the development of EMT and the onset of hepatocellular carcinoma. E3 ligase TRIM26 also degrades ZEB1 through ubiquitination and inhibits cell growth, development, and metastasis of HCC. USP39 and TRIM26 bind to each other directly and maintain the ubiquitin level of ZEB1, thus determining the proliferation and migration of HCC (Li X. et al., 2021).

### 2.2 The negative regulatory KLF17

KLF17, SP/KLF zinc finger protein family, inhibits EMT and metastasis of cancer. The DNA binding domain (DBD) or C2H2-ZF domain on its C-terminal of KLF17 binds to the relevant G/C box and CACCC box of the gene and inhibits transcription (Sun et al., 2013; Lea et al., 2021). Ali and colleagues have found a new mechanism of interaction between TGF-β/Smad-KLF17 axis in the progress of tumor metastasis. KLF17 promotes TGF/Smad-dependent signaling to prevent tumor development. At the same time, it was also found that TGF/Smad3 signaling enhances the expression of KLF17, resulting in a positive feedback loop (Ali et al., 2015). Sun found that miR-9 downregulated the expression of KLF17 protein through directly binding to KLF17 on its 3′-UTR region, resulting in up-regulating EMT-related genes (ZO-1, Vimentin, and Fibronectin (FN)) and promoting HCC migration and invasion (Sun et al., 2013).

## 3 CSCs in HCC

Cancer stem cells (CSCs) are a category of cancer cells. The ability of CSCs to escape cell death and metastasis is of great significance to tumorigenesis (Plaks et al., 2015; Batlle and Clevers, 2017). The increased transcription of pluripotency-associated factors (including SOX2, c-myc, BMI1, Nanog, etc.) in CSCs is related to tumor progression, chemotherapy resistance, and recurrence (Wang et al., 2010; Sekiya and Suzuki, 2011; Shan et al., 2012; Ladewig et al., 2013; Zhang et al., 2016; Novak et al., 2020). C2H2-ZF protein could bind to pluripotency-associated factors. As a result, numerous transcription factor binding loci (MTL) are formed. In addition, most of the surface markers (such as CD13, CD24, CD44, CD90, CD133, and EpCAM) are connected to the incidence and progression of malignancies in HCC (Yang et al., 2008; Yamashita et al., 2009; Yang et al., 2009; Haraguchi et al., 2010; Lee et al., 2011; Tang et al., 2012; Kim and Park, 2014; Yan et al., 2015; Batlle and Clevers, 2017). C2H2-ZF protein regulates the mRNA or protein expression of these markers and promotes growth and proliferation through its regulatory actions on liver CSCs. Therefore, investigating the transcription regulatory mechanisms of the self-renewal and pluripotency of CSC is helpful to HCC patients.

### 3.1 ZNF687

ZNF687 directly binds the promoter and increases the transcriptional expression of pluripotency-related factors (BMI1, Oct4, and Nanog), promotes the tumorigenesis of HCC cells *in vivo*, and increases the HCC SP+/CD133+ populations *in vitro*. CD133 regulates the incidence and growth of CSCs in HCC via NTS, IL-8, CXCL1, and MAPK signals (Tang et al., 2012). This study also found that the interaction between ZNF687 and the activity enhancer MBD3/NuRD complex promotes Nanog-induced pluripotency (Kloet et al., 2015), suggesting that ZNF687 has been linked to transcription activation and cell reprogramming regulation. Nanog-positive cells show intensive self-renewal, clone formation, tumor initiation, resistance to therapeutic drugs such as sorafenib and cisplatin, and a strong ability for tumor migration (Shan et al., 2012). At the same time, it was found that ZNF687 may induce the upregulation of pluripotency-related factors through the Wnt/β-catenin signal pathway (Zhang et al., 2017).

### 3.2 ZEB1

Tsai and colleagues reported that the miR-200b-ZEB1 circuits are linked to the initiation and development of various CSCs. ZEB1 binds to the promoters of surface markers, causing CD13 and CD24 to be upregulated and EpCAM to be downregulated. CD13 can reduce DNA damage induced by ROS and protect cells from apoptosis (Haraguchi et al., 2010). Through STAT3-mediated Nanog overexpression, CD24^+^ hepatoma cells activate tumor development and self-renewal (Yamashita et al., 2009; Lee et al., 2011). EpCAM is a biomarker of hepatic stem cells (HPSCs), which activates the Wnt/β-catenin signals and maintains the characteristics of HSPCs and also can activate c-Myc, Oct3/4, SOX2, and KLF4 to induce fibroblasts to differentiate into pluripotent stem cells in HCC (Yamashita et al., 2009; Kim and Park, 2014). MicroRNA-200b directly inhibits the expression of BMI1 and ZEB1. BMI1 gives cells the ability to renew themselves and promotes cell growth and development, colony formation, invasion, and metastasis (Zhang et al., 2016). Downregulation of miR-200b combined with overexpression of ZEB1 promotes the creation and maintenance of CD13+/CD24+/EpCAM-CSCs. In contrast, upregulation of miR-200b combined with downregulation of ZEB1 leads HCC cells to EpCAM + CSCs. Downregulation of miR-200b was linked to tumor recurrence and prognosis, suggesting that the miR-200b/ZEB1 axis is critical in the formation and maintenance of CSCs for tumorigenesis, relapse, and chemotherapy resistance. (Tsai et al., 2017).

### 3.3 ZFX

Zinc finger protein X-linked (ZFX) encodes on the mammalian X chromosome and contains a DBD with 13 C2H2-ZF at the 3′-end (Schneider-Gädicke et al., 1989). Lai and colleagues found that ZFX binds to Nanog and SOX2 and therefore activates their transcription in HCC and promotes tumor growth. SOX2 contributes to cancer progression and augments therapy resistance in HCC (Novak et al., 2020). On the contrary, the knockout of ZFX can cause the G0/G1 cell cycle to halt, decrease the ability of cell proliferation and colony formation of liver cancer cells, and increase the sensitivity of HCC cells to chemotherapeutic drug cisplatin (Lai et al., 2014). Wang found that ZFX activates and maintains the cell stem of EpCAM + CSCs through the Wnt/β-catenin signal. ZFX promotes the nuclear transcription of *β*-catenin, enhances its transcriptional activity, and induces the expression of downstream genes such as CylinD1, thus promoting the growth and proliferation of liver stem cells (Wang et al., 2017).

### 3.4 ZBP-89

ZBP-89 (also called ZNF148), as a tumor suppressor, can suppress the development of liver cancer in a variety of ways. In patients with HCC, the overexpression of ZBP-89 may indicate improved survival and decreased recurrence (Wang et al., 2018). Wang and colleagues have found that ZBP-89 downregulates the stem cell-like characteristics in HCC by inhibiting Notch1 signals. ZBP-89 locates and binds to Notch1 intracellular domain (NICD) in the nucleus, competitively blocks the NICD transcriptional activator complex, and inhibits the expression of EpCAM and CD44 in a dosage-dependent manner, which has a negative regulatory effect on liver cancer stem cells. CD44 is also found to have a critical player that is a key regulator of CSC features such as self-renewal, tumorigenesis, invasion, and drug resistance (Yan et al., 2015). At the same time, they also found that NOTCH1 may suppress ZBP-89 and form a negative feedback loop to sustain cancer cell existence (Wang et al., 2020).

### 3.5 ZHX2

Zinc finger and homeboxes 2 (ZHX2) was initially identified to regulate the oncofetal gene AFP, H19, and GPC3 in mice, showing that it is involved in HCC. Lin and colleagues have proved that ZHX2 plays a tumor-inhibitory role in liver cancer. ZHX2 inhibits the demethylation of histone H3 lysine36 (H3K36) in the promoter region of stemness-related transcription factors (including SOX2 and Oct4) mediated by lysine demethylase 2A (KDM2A), and suppresses liver CSCs activity and sorafenib resistance. The overexpression of Oct4 induces the activation of TCL1 and AKT to mediate chemotherapy resistance. The Oct4-TCL4-AKT axis affects the proliferation of embryonic stem cells and CSCs by inhibiting apoptosis (Wang et al., 2010). In addition, there is a negative association between the expression of ZHX2 and KDM2A in human HCC, and high ZHX2 expression and low KDM2A expression are associated with the prognosis in HCC (Lin et al., 2020).

## 4 Metabolic reprogramming in HCC

Metabolic reprogramming is a marker of malignant tumors, affecting the growth, development, and tumor microenvironment in HCC (Sun et al., 2018). In the occurrence and development of HCC, liver cancer cells could obtain energy and biomass synthesis to gain support for cell survival, and escape immune surveillance and proliferative growth by reprogramming their catabolism and anabolism (Guo et al., 2014). Metabolic changes are accompanied by tumor progression and metastasis and diffusion of cancer cells, which require nutritional absorption and biosynthesis in the early stage of tumor growth and depend on new ways to promote tumor metastasis, invasion, and chemical drug resistance in the advanced stage of HCC (Faubert et al., 2020). The metabolic interaction between tumor cells and the tumor ecosystem will promote therapeutic resistance (Schulze and Harris, 2012), so the metabolic changes of cancer cells and the mutual degradation of the microenvironment become another key mechanism to regulate cancer progression.

### 4.1 Aerobic glycolysis

In cancer cells, when glycolysis flux is upregulated, pyruvate can produce lactate under oxygen-rich environments, a process called the Warburg’s effect or aerobic glycolysis (Koppenol et al., 2011; Bensaad and Harris, 2014). Many cancers, including liver cancer, are marked by enhanced glucose absorption and glycolysis, suggesting that metabolic changes provide a growth advantage for tumor cells (Schulze and Harris, 2012). Cancer cells promote the production of ATP and lactic acid buildup, regulate the pH value of the tumor ecosystem, and affect the signal pathway via Warburg’s effect, which offers cancer cells selective benefits in terms of growth, continued existence, migration, and metastasis (Lunt and Vander Heiden, 2011; Teoh and Lunt, 2018).

#### 4.1.1 PKM2

Zinc finger protein acts on the key enzyme regulating the rate-limiting stage in glycolysis, and promotes or inhibits the aerobic glycolysis of tumor cells by up-regulating or inhibiting the rate-limiting enzyme. For example, there are two isoforms of pyruvate kinase M(PKM). PKM1 is abundant in mature cells, which increases ATP production, while PKM2 is commonly abundant in embryos and cancer tissue, which boosts the Warburg’s effect of HCC (Yang and Lu, 2013). The expression of PKM2 offers cancer a selective survival benefit. PKM2 participates in the metabolism of cancer reprogramming, and promotes tumor growth and development, which prompt people to study the metabolic and regulatory process behind the role of PKM2 in HCC (Benjamin Daniel et al., 2012).

#### 4.1.2 ZFP91

Past research has discovered that E3 ligase ZFP91 interacts with hnRNP A1, a splicing regulator, to boost proteasome degradation of hnRNP A1. As a result, hnRNP A1-dependent PKM mRNA pre-mRNA splicing is inhibited, causing PKM1 isoform formation while inhibiting PKM2 isoform formation. The results show that ZEP91 inhibits liver cancer cells growth and proliferation, which participates in ubiquitination and catabolism of substrates, and suppresses metabolic programming, cell growth, and development of liver cancer (Chen et al., 2020).

#### 4.1.3 ZEB1

The latest study found that silencing ZEB1 in different HCC cell lines substantially reduced levels of protein and aerobic glycolysis of the muscle isoform of phosphofructokinase-1 (PFKM), another rate-limiting enzyme in glycolysis, which was characterized by a decrease in glucose uptake and lactic acid production. The exogenous expression of PFKM significantly reduced this decline. Further study found that ZEB1 interacts with the non-classical ZEB1 binding motif in the PFKM promoter region, thus activating its transcription (Zhou et al., 2021). It is suggested that a new mechanism of direct relationship between zinc finger protein and promoting glycolysis and Warburg effect in the development of HCC.

### 4.2 NAFLD-NASH-HCC

In addition to glucose, the increase in lipid synthesis has been considered a component of cancer cell metabolic reprogramming (Schulze and Harris, 2012). Lipids synthesized by tumor cells are important components of biofilm lipids and important substrates of energy metabolism and produce lipid signal molecules to promote tumor proliferation and metastasis (Menendez and Lupu, 2007). Non-alcoholic fatty liver disease (NAFLD) and non-alcoholic steatohepatitis (NASH) could accelerate liver fibrosis, cirrhosis, end-stage liver disease, and eventually develop into liver cancer, indicating that lipid metabolism may be a determining factor in the development of NAFLD to HCC (NAFLD- NASH-HCC) (Schuppan and Schattenberg, 2013; Jiang et al., 2014). In recent years, some zinc finger proteins have been shown to transcribe key enzymes or lipid metabolism-related genes and proteins and affect the progression of NAFLD-NASH-HCC (Wu et al., 2020; Yu et al., 2020).

#### 4.2.1 ZHX2

Lipoprotein lipase (LPL) is a key enzyme in lipid metabolism, which encourages the development and proliferation of liver cancer through the uptake of exogenous lipids. It has been found that LPL is the target of ZHX2. In NAFLD, ZHX2 inhibits NAFLD-HCC progression and cell growth retardation through transcriptional inhibition of LPL expression. In HCC cells, ZHX2 inhibits LPL and further suppresses the proliferation, lipid deposition, growth, and formation of exogenic and spontaneous tumors (Wu et al., 2020). Further studies found that ZHX2 boosted the transcription of miR-24–3p, which aimed at SREBP1c and caused its destruction. Moreover, ZHX2 inhibited *de novo* lipogenesis (DNL) and the development and transfer of HCC (Wu et al., 2020).

#### 4.2.2 SREBP1c

Sterol-responsive element-binding protein 1c (SREBP1c) is an important TF for DHL and is an important link between tumor signal transduction and tumor metabolism (Lee et al., 2019). SREBP1c participates in the production of cholesterol and fatty acids and has been proven to be overexpressed in HCC (Guo et al., 2014). Tumor cells reactivate DNL to provide nutrition for tumor cells, which is conducive to tumor cell growth and tumor symbiosis (Schulze and Harris, 2012). Therefore, zinc finger protein can delay the progression from NAFLD to HCC and the growth and invasion of HCC via transcriptional control of key regulatory factors in DNL.

## 5 Cell cycle in HCC

Cell cycle disorder can contribute to excessive cell division, and sustaining proliferative signaling is one of the markers of various malignant tumors (Hanahan and Weinberg, 2011). Cell cycle proteins and cell division cycle proteins are required for cell cycle precise control.

### 5.1 ZNF384

In the G1 phase, cyclin-dependent kinases (CDK) 4 and 6 bind to one of the three D-type cyclins (D1, D2, D3) in different environments and promote cell cycle transition after transcriptional induction (Malumbres, 2014; Ingham and Schwartz, 2017). C2H2-ZF protein 384 (ZNF384) upregulates the expression of CyclinD1 by recognizing the adenine-rich region of the promoter of CyclinD1. The interaction between CyclinD1 and CDK4 or CDK6 promotes the G1/S stage transformation and the multiplication in liver cancer (He et al., 2019). Cell division cycle 6 (CDC6) regulates DNA replication in mammalian cells, which regulates DNA replication or checkpoint mechanism (Karanika et al., 2017).

### 5.2 ZNF143

CDC6 participates in the assembly of the complex before replication and promotes the G1/S phase transition (Borlado and Mendez, 2008). The DNA over-replication found in cancer cells following the ectopic expression of CDC6 (Vaziri et al., 2003). C2H2-ZF protein 143 (ZNF143) promoted the expression of CDC6 by directly activating the transcription of histone demethylase mineral dust-induced gene (MDIG), which reduced H3K9me3 enrichment in the CDC6 promoter region and promoted the cell cycle progression of HCC (Zhang et al., 2020).

### 5.3 ZNF233

In addition, C2H2-ZF protein 233 (ZNF233), the Krüppel C2H2-ZF family, accelerates growth and proliferation by promoting the G1/S phase transition in HCC cells. Clinical studies have shown that the level of expressiveness of ZNF233 is associated with the tumor grade, stage, and clinical presentation of patients with HCC (Xie et al., 2018).

## 6 Anoikis in HCC

Anoikis, a type of apoptosis that occurs when epithelial or endothelial cells detach from the extracellular matrix, is also another form of “loss-of-signal"-induced cell death (ECM). Unligated ECM proteins of the integrin family give up to stimulate pro-survival signaling under this circumstance, ultimately causing cell death (Green and Llambi, 2015). Focal adhesion kinase (FAK) is a common and important tyrosine kinase that transduces signals from integrins, growth, and endocrine elements and is involved in many essential biological functions and activities (Franchini, 2012). Src family kinases, a player in promoting metastasis in many tumor types, are critical components of the anoikis resistance pathway (Dai et al., 2016). Src recruits FAK and promotes Src phosphorylation (Acebron et al., 2020). The activation of Src activates downstream target protein kinase AKT and regulates cell resistance to apoptosis (Dai et al., 2016). As a receptor of intracellular ROS level, C2H2-ZF protein 32 (ZNF32) maintains redox homeostasis and promotes the resistance of HCC to anoikis through inhibiting excessive ROS accumulation of HCC and activating Src/FAK signal pathway (Li et al., 2019).

## 7 Genomic integrity in HCC

Because genomic dysfunction affects many other human diseases in addition to cancer and aging (Sarek et al., 2015). It is essential to understand the regulation and maintenance of the genome, including DNA damage response (DDR) and telomere maintenance (Jackson and Bartek, 2009). Zinc finger domains are found in some DNA damage factors and telomere-associated proteins. Zinc finger domains have exceptional binding flexibility and may attach to Nucleotides, molecules, and protein post-translational modifications (PTMs), which are involved in genome integrity (Gamsjaeger et al., 2007).

### 7.1 DNA damage response

#### 7.1.1 ZNF451

ZNF451 is a class of SUMO E3 ligases and a multifunctional DDR factor, containing two N-terminal SIM domains and a ubiquitin-interacting motif. Extensive biochemical analysis shows that the C2H2-ZF domains and tandem SIM domains are the keys to its E3 ligase activity (Cappadocia et al., 2015; Eisenhardt et al., 2015; Schellenberg et al., 2017). ZNF451 and Topoisomerase 2 (TOP2) are involved in DDR and break the DNA double helix and topological changes (Deweese and Osheroff, 2009; Schellenberg et al., 2017), which could affect DNA supercoil formation during transcription and replication, and lead to genomic instability (Ju et al., 2006; Pommier, 2013; Tammaro et al., 2013; Madabhushi et al., 2015). Shanbhag found that DNA double-strand breaks (DSB) cause extensive local and global changes in chromatin structure, many of which depend on ATM kinases, leading to transcriptional silencing (Shanbhag et al., 2010). DNA double-strand break (DSB), one of the main types of DNA damage, promotes cell cycle halt and DDR to maintain the integrity of the genome via activating a series of cell activities.

#### 7.1.2 ZNF143

CDC6 can protect the integrity of the genome via activating DDR (Yoshida et al., 2010), but overexpression of CDC6 may lead to repetitive replication and genomic instability, which is essential for tumor progression (Liontos et al., 2007). Zhang and colleagues proposed a new pathway of ZNF143-MDIG-CDC6 regulating in liver cells (Zhang et al., 2020). In addition, Understanding the interplay between transcription and DNA repair mechanisms will give critical insights and, eventually, novel therapeutic options for a variety of disorders linked with genome maintenance abnormalities (Sebastian and Oberdoerffer, 2017).

### 7.2 Telomere maintenance

In mammalian cells, the maintenance of the Chromatin structure promotes the self-renewal of human embryonic stem cells (HESCs) and induced pluripotent stem cells (HiPSCs) (Blasco, 2007; Huang et al., 2011; Pucci et al., 2013; Dan et al., 2014; Rivera et al., 2017). Telomere erosion and telomere lengthening mechanisms that are two opposite forces determine the length of the telomere.

#### 7.2.1 Zscan4

C2H2-ZF protein has become a key controller of telomere length. Dan has shown that zinc finger and SCAN domain containing 4 (Zscan4) inhibits telomere extension linked with the expression of Zscan4 by recruiting Uhrf1 and Dnmt1 (the main component of DNA methylation mechanism) and promoting their degradation, blocking DNA demethylation (Dan et al., 2017).

#### 7.2.2 ZNF827

ZNF827 is abundant in telomeres and uses the ALT pathway to recruit DNA repair factors, which encourage homologous recombination and telomere lengthening. Conomos believe that ZNF827 attachment to human telomeres induces telomeric chromatin modification and the formation of an atmosphere that encourages telomere-telomere recombination, as well as the integration and regulation of numerous molecular components of ALT function (Conomos et al., 2014).

#### 7.2.3 ZBTB48

ZBTB48 (also known as HKR3 or TZAP) has 11 C2H2-ZF domains, three of which interact with TTAGGG motifs specifically. It was found that ZBTB48 binds to double-stranded TTAGGG repeats and acts as a negative regulator on telomeres (Li J. et al., 2017; Jahn et al., 2017; Kappei et al., 2017). When ZBTB48 is located at telomeres, a process called telomere trimming is triggered, resulting in the rapid deletion of telomere repeat sequences (Li J. et al., 2017). Due to the phenomenon that telomere maintenance is commonly and initially changed in HCC, telomerase targeting is a promising anti-cancer strategy.

## 8 Anti-tumor drug resistance in HCC

**FIGURE 4:**
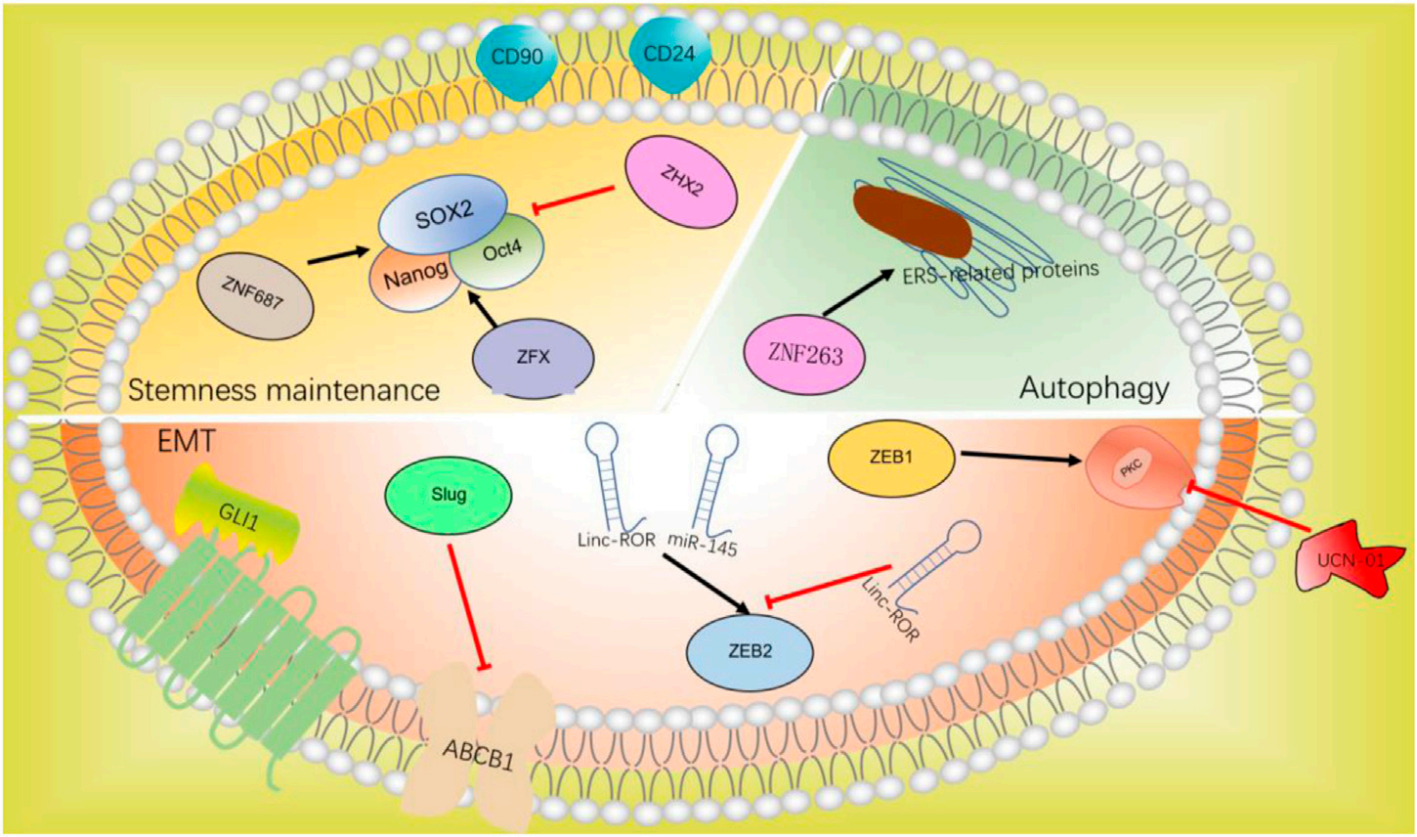
C2H2-ZF protein in chemotherapeutic resistance.

HCC is distinguished by a high level of inherent chemoresistance, resulting in a restricted effect of chemotherapy and recurrence since therapy. Multidrug resistance is one of the causes of poor cure rates in advanced HCC (Khaled et al., 2022). Zinc finger proteins are not only taking part in the initiation, growth, progression, and metastasis of HCC but also in drug resistance to chemotherapy. Zinc finger proteins regulate the resistance of chemotherapeutic drugs and targeted drugs through various mechanisms and pathways. According to the research in recent years, we will elaborate on the regulation of C2H2-ZF protein in chemotherapeutic resistance from three aspects of EMT, stemness maintenance, and autophagy (Figure 4).

### 8.1 EMT and anti-tumor drug resistance

The role of EMT in anti-tumor drug resistance has been more and more recognized. C2H2-ZF proteins such as ZEB1, ZEB2, Slug, and GLI1 participate in the progression of EMT can also promote anti-tumor drug resistance.

#### 8.1.1 ZEB1

ZEB1-induced EMT can lead to resistance to conventional chemotherapy. ZEB1 can classify hepatocellular carcinoma into epithelial type and mesenchymal type *in vivo* and *in vitro*. Mesenchymal/metastatic cancer cells need to activate the protein kinase C (PKC) pathway to survive (Singh et al., 2009). Sreekumar and colleagues have demonstrated that the PKC signal is stimulated in mesenchymal HCC cells, which helps them proliferate. Mesenchymal cells and chemotherapy-resistant cells that express ZEB1 are dependent on PKCa, which can be eliminated selectively by PKC inhibitor UCN-01. The inactivation of PKCa also reduces the activity of HCC, showing that UCN-01 in conjunction with chemotherapy can improve the therapeutic effect of ZEB1-positive HCC (Sreekumar et al., 2019). LONG studies have shown that the upregulation of ZEB1 expression is associated with the EMT characteristics of HCC, while inhibition of ZEB1 expression makes cells sensitive to chemotherapy (Long et al., 2019). Targeted inhibition of the ZEB1 gene may contribute to clinical chemotherapy resistance.

#### 8.1.2 ZEB2

ZEB2 has a significant impact on the progression of HCC by regulating EMT. The interaction between Linc-ROR and miR-145 upregulates the transcription of ZEB2, which promote the proliferation and metastasis of HCC cells (Li C. et al., 2017). Recent research found that miR-212–3p reduces the transcription of ZEB2 and reduces the EMT, migration, and metastasis of HCC by targeting ZEB2, thus reducing paclitaxel resistance, which provides a new target for liver cancer chemotherapy (Yang et al., 2020).

#### 8.1.3 SNAI2/Slug

Snail family transcriptional repressor 2 (SNAI2/Slug), belongs to the part of the Snail family of C2H2-ZF TFs and is a transcriptional repressor that interacts with E-box domains (Hajra et al., 2002). Slug is the key regulator of EMT, inducing EMT and inhibiting E-cadherin in various epithelial cells (Shih et al., 2005; Uchikado et al., 2005). Previous studies have shown that Slug inhibits multidrug resistance by inhibiting ABCB1 *in vivo* and *in vitro*, thus exerting tumor inhibitory effect (Hoshida et al., 2016). In contrast, recent research has shown that ABCB1 is downregulated in HCC cells undergoing EMT, while chemotherapy resistance to cisplatin has more than doubled. It is concluded that the chemotherapy resistance of HCC is sometimes influenced by changing Slug. The expression of Slug could affect chemotherapy resistance, depending on the cell environment (Karaosmanoglu et al., 2018).

#### 8.1.4 GLI1

Hedgehog (HH) signaling pathway is closely related to cancer growth and differentiation. Abnormal HH signal has been considered to be an important signal pathway and therapeutic target for human tumors (Lum and Beachy, 2004). HH signal is closely related to many basic processes, and the imbalance of this signal axis may lead to tumor occurrence, metastasis, and drug resistance. HH signaling has been found to promote tumor development at the molecular level by influencing tumor growth, EMT, CSC development, and acquired drug resistance (Ochoa et al., 2010; Sari et al., 2018). C2H2-ZF protein GLI1, the terminal effector of HH signaling, is a potential marker of signaling activation because it promotes gene expression. Chen and colleagues have found that EMT and elevated HH signaling activation may be causes of chemical resistance and invasion in poorly differentiated HCC cells (Chen et al., 2011). Further study showed that the protein level of GLI1 in poorly differentiated HCC cells was significantly increased. After the decrease of GLI1 expression, the responsiveness of HCC cells to doxorubicin and cisplatin increased significantly. Targeted HH signaling pathway gives a fresh viewpoint on drug resistance in the treatment of HCC (Zhou et al., 2020).

### 8.2 Stemness maintenance and anti-tumor drug resistance

Stemness maintenance is related to tumor recurrence and chemotherapy resistance (*vide supra*), in which the high expression of Nanog and Oct4 in CSCs promotes chemotherapy resistance, and the surface markers CD24 and CD90 are related to chemotherapy resistance in HCC.

#### 8.2.1 ZNF687

ZNF687 upregulates pluripotency-related factors. Overexpression of ZNF687 made cisplatin-treated liver cells resistant to apoptosis, and silencing ZNF687 significantly enhanced the sensitivity of HCC to cisplatin. These results indicate that ZNF687 has a significant impact on the chemical resistance of HCC (Zhang et al., 2017).

#### 8.2.2 ZFX and ZHX2

Zinc finger protein X-linked (ZFX) binds to the promoters of Nanog and SOX2 to promote tumor growth. In contrast, the knockout of ZFX increases the chemotherapeutic responsiveness to HCC (Wang et al., 2017). ZHX2 inhibited the demethylation of SOX2, Nanog, and Oct4, and inhibited the chemical resistance of liver cancer (Lin et al., 2020).

### 8.3 Autophagy and anti-tumor drug resistance

Autophagy is a critical biological function that influences cell metabolism, proteome, quantity, and quality of organelles, and changes cell function in different ways (Kimmelman and White, 2017). In cancer, it can inhibit the tumor or increase survival under the pressure of treatment (chemotherapy and targeted drugs), thereby increasing treatment resistance (Thorburn et al., 2014; Singh et al., 2018). Continuous exposure to sorafenib can activate endoplasmic reticulum stress (ERS)-related proteins, as well as induce autophagy and sorafenib resistance. Inhibition of ERS can reduce autophagy and reverse sorafenib resistance (Cheng et al., 2020). ZNF263 is an ERS-specific transcription factor and the downregulation of its expression reduces chemotherapy resistance and increases apoptosis rate. Knockout of ZNF263 effectively alleviates the chemotherapy resistance of ERS-related autophagy-induced HCC cells (Cui et al., 2020). The study of ERS-specific transcription factors provides a different strategy to deal with drug resistance: inhibition of autophagy to avoid resistance to kinase inhibitors such as sorafenib (Amaravadi et al., 2016).

## 9 Conclusion

C2H2-ZF proteins are abnormally expressed in HCC and involve in numerous HCC features like EMT, stemness maintenance, metabolic reprogramming, cell proliferation and growth, apoptosis, and genomic integrity. These proteins are also correlated with and regulate chemoresistance through targeting and modulating the activities of key molecules involved in these processes, such as EMT, stemness maintenance, and autophagy. However, the regulatory mechanisms of C2H2-ZF proteins in HCC are still unclear and the related studies are also still in the initial stage. This paper summarizes the regulatory role of C2H2-ZF proteins in hepatocarcinogenesis and progression as follows: 1. C2H2-ZF proteins play an important role in regulating the transcription of downstream genes that are involved in the proliferation, apoptosis, migration, and invasion of HCC (Liang et al., 2017). 2. The differential expression levels of C2H2-ZF proteins in HCC are regulated by tumor-associated miRNAs (Li C. et al., 2017). 3. Involvement in post-translational modifications through ubiquitination, a modality that can increase zinc finger protein transcriptional activation or inhibit post-translational regulation (Dong X. et al., 2021). 4. Zinc finger proteins show different sequence specificity in their ability to bind DNA, and the diversity of zinc finger proteins is demonstrated after different combinations of zinc finger motifs (Dan et al., 2017). 5. Transcriptional regulation is an extremely complex biological process, and the microenvironment within tumor cells may be an important factor in addition to the structural diversity of the same C2H2-ZF protein, which results in multiple transcriptional regulation patterns (Zhou et al., 2021). Therefore, the role of C2H2-ZF protein in regulating different molecules, signal pathways, and pathophysiological activities shows that C2H2-ZF protein could be used as diagnostic or prognostic markers and could be considered as potential therapeutic targets or sensitizers in HCC. We believe that C2H2-ZF proteins would have a broad application prospect in the diagnosis and drug treatment of HCC with the continuous improvement of the detection, verification, and functional analysis of them. However, more studies demand to pave the way for the clinical application of these findings.
